# The Role of Family Life and the Influence of Peer Pressure on Delinquency: Qualitative Evidence from Malaysia

**DOI:** 10.3390/ijerph19137846

**Published:** 2022-06-26

**Authors:** Ezarina Zakaria, Noor Nasihah Kamarudin, Zhooriyati Sehu Mohamad, Masahiro Suzuki, Balan Rathakrishnan, Soon Singh Bikar Singh, Zaizul Ab Rahman, Vikneswaran Sabramani, Azianura Hani Shaari, Mohammad Rahim Kamaluddin

**Affiliations:** 1 Centre for Research in Psychology and Human Well-Being, Faculty of Social Sciences and Humanities, The National University of Malaysia, Bangi 43600, Selangor, Malaysia; noornasihakamal91@yahoo.com (N.N.K.); azianura@ukm.edu.my (A.H.S.); 2 Faculty of Social Sciences and Liberal Arts, UCSI University Kuala Lumpur (South Wing), Cheras, Kuala Lumpur 56000, Selangor, Malaysia; zhooriyati@ucsiuniversity.edu.my; 3 School of Business and Law, Central Queensland University, Melbourne, VIC 3000, Australia; m.suzuki@cqu.edu.au; 4 Faculty of Psychology and Education, Universiti Malaysia Sabah, Kota Kinabalu 88400, Sabah, Malaysia; rbhalan@ums.edu.my (B.R.); soonbs@ums.edu.my (S.S.B.S.); 5 Research Centre for Theology and Philosophy, Faculty of Islamic Studies, The National University of Malaysia, Bangi 43600, Selangor, Malaysia; zaizul@ukm.edu.my; 6 Malaysian Association of Adolescent Health, Bukit Jalil Integrated Business Park, Kuala Lumpur 58200, Selangor, Malaysia; drvikneswaran@yahoo.com

**Keywords:** young male prisoners, delinquency, parenting, peer pressure, Malaysia

## Abstract

Juvenile delinquency is always seen as a public health problem which needs intervention at various levels. Identifying which factors may lead juveniles to delinquency is a long-standing question among criminologists. This remains the case in Malaysia. There are studies that have explored the impact of problem-solving skills, low socioeconomic status, and gender differences in predicting the delinquent behavior of youth in Malaysia. However, very few studies have aimed to find an in-depth understanding of the effects of family roles and peer pressure on delinquency in Malaysia. The present qualitative research was designed to fill this gap in the literature. In-depth interviews were conducted with 12 young male prisoners (juvenile delinquents) in Malaysia to explore the influences of family life and peer pressure on delinquency. The current study showed that parental un-involvement, parent separation, peer pressure, criminal gang membership, and parents’ involvement in crime were the important factors for involvement in delinquency. The findings revealed the importance of guidance and counseling for parents and adolescents, to help them cope with life challenges and to build their social and emotional skills, as well as the necessity of appointing school psychologists and public health experts to help the youths become valuable individuals.

## 1. Introduction

Since time immemorial, family has been considered to be a vital element in a child’s development. Taking care of children is the primary responsibility of parents, and the values they give determine the children’s behavior and lifestyle when they reach adulthood [1]. Parents are the ones who should teach emotional skills to their children. A classic study found that children who lack emotional skills often engage in criminal activities [2]. Marital conflict also determines the future success of the child, as the conflicts influence the child’s development [3]. Healthy development of the child includes emotional, intellectual, and social development.

Families fail to function properly due to a variety of problems, such as family members being involved in substance abuse, being in detention, having divorced parents, having mental and/or physical health problems, domestic violence, low socioeconomic status, low levels of education, religion, poor parenting skills, and so on. These situations can cause families to fail to create a prosperous environment for adolescents [4]. As a result of the family’s dysfunction, the children’s education is also neglected, causing the problem of adolescents dropping out of the formal education system, namely school. If the family aspect fails to be given well due to various factors, such as troubled parents, divorce, death, and so on, and no other people (for example, other immediate family members, local community members, etc.) take over the task, then the risk is that adolescent development will not progress well, and the adolescent may exhibit delinquent behavior.

On another note, adolescence is also the period in which individuals like to spend most of their time with their peer group, as peer groups offer a better environment in terms of equality, excitement, and freedom. Acceptance from the peer group is also considered critical during this period. For acceptance, adolescents will tend to do anything, without carefully thinking about the consequences of their behavior. However, rejection and neglect by peers are related to subsequent mental health and criminal issues for an individual [5].

The nation requires healthy individuals for its growth. Youngsters are future leaders who will determine the development and well-being of a country. Therefore, young people need to ensure that they excel in various aspects of life, such as in academics, employability skills, personal development, and other qualities which are essential for enhancing their capabilities and potential as future leaders. To realize this, primary preventions are crucial. We must identify the potential risk factors and implement timely sustainable measures to eliminate those hazards. Thus, the harmonious growth of mind and soul will be gained. Nevertheless, from the perspective of public health, strong leadership with a clear vision is fundamental in ensuring all objectives are met.

However, this effort is not an easy task, because unlimited exposure to the world brings challenges and pressures to the younger generation when coping with various demands in life. One of the challenges that can delay this effort would be the participation in risky behaviors, including delinquency and crime among the younger generation. Given the lack of information on the role played by the family and peers in the emergence and persistence of delinquent behavior in adolescents in Malaysia, we seek to identify in the accounts of the participants (1) the value assigned to the family experience in the initiation and maintenance of this behavior, and (2) the influence exerted by peers in its development. The aim of the study is to generate contextualized evidence in Malaysia regarding two of the dynamic risk factors most widely reported in the international scientific literature.

### 1.1. Juvenile Justice System in Malaysia

The principal Act governing the handling of children, including juveniles in conflict with the law in Malaysia, is the Child Act 2001 (Act 611), which came into force in August 2002. This Act consolidated three former Acts, which were being used before the introduction of the current Act 611. They were, namely, the Juvenile Courts Act 1947 (Act 90), the Women and Girls Protection Act 1973 (Act 106), and the Child Protection Act 1991 (Act 468). The current Child Act of 2001 governs four main categories of children: (1) children in need of care and protection; (2) children in need of protection and rehabilitation; (3) children “beyond control”; and (4) children in conflict with the law.

The Malaysian Child Act 2001 outlines the main structure, processes, and procedures in place for responding to children who commit criminal offenses. Part X of the Act stipulates special procedures for arrest, bail or remand, trial, and sentencing of children, and defines the roles and responsibilities of police, probation officers, the Court for Children, and various other institutions that handle child offenders. Pursuant to section 83(1) of the Act, a child who is arrested, detained, and tried for any offence (subject to certain specified limitations) must be handled in accordance with the provisions of the Child Act, rather than the standard criminal procedures applicable to adults. The special procedures under the Child Act modify and take precedent over any written laws relating to procedures for arrest, detention, and trial. However, where the Child Act does not address a specific issue, then reference may be made to the standard criminal procedures under the Malaysian Criminal Procedure Code (Act 593). Section 11(6) of the Child Act 2001 clearly states that “Except as modified or extended by this Part, the Criminal Procedure Code shall apply to Courts for Children as if Courts for Children were Magistrates’ Courts”.

In 2021, the Department of Statistics Malaysia reported that the number of children involved in crime in 2020 had increased by 10.5% to 5342 cases, compared to 2019 (4833 cases). From this percentage, first offence cases increased by 15.7% to 4916 cases, whereas repeated offences recorded a decrease of 27.2% to 426 cases, compared to 2019 [6].

Malaysia has outlined procedures, rules, and regulations in place to deal with juvenile offenders. Malaysia has a court that specifically deals with children and juvenile offenders. The Children’s Court in Malaysia was established in the Child Act 2001. The word “child” was defined under the Child Act 2001 as (a) a person below the age of eighteen years and, (b) in criminal proceedings, a person who has attained the age of criminal responsibility following section 82 of the Penal Code. Under section 97 of the Child Act, a Children’s Court should not allow a death sentence if the crime was committed by a child. However, section 11(5) of the Child Act 2001 only permits a Children’s Court to try the child for offences that are not punishable by death. If there are cases that involve the death penalty, they will be handled outside the jurisdiction of the Children’s Court. Detention at the Henry Gurney School is one of the punishments executed by the Children’s Court. The Henry Gurney School is a rehabilitative school prepared for convicted young offenders in Malaysia.

The Children’s Court can send a child to the Henry Gurney School (under section 75 of the Child Act) (1) if the child is found guilty and the offence is punishable with imprisonment, and (2) if the probation report indicates that the parents (or guardians) of the child cannot or are not supervising the child’s behavior. The Children’s Court will also send a child to the Henry Gurney School if the court feels that the offence is serious, and that the detention can reduce the child’s tendency to commit criminal activities in the future.

Under section 293 of the Criminal Procedure Code (CPC), a court may punish “youthful offenders” by

1. Ordering the offender to be discharged, after giving a stern warning (“due admonition”);
2. Ordering the offender to be delivered to his nearest relative, or parent, and making him execute a bond for twelve months of good behavior;
3. Ordering the offender (male only) to be whipped by a cane for up to 10 strokes;
4. Dealing with the offender following provisions of the Child Act 2001; or
5. Ordering the offender to carry out community service, of up to 240 h.

However, the most important aspect that determines the punishment is the age of the offender. In Malaysia, the juvenile offender’s age classifies whether he or she is a “child” under the Child Act 2001, or a “youthful offender” under the Criminal Procedure Code. The age classification is simplified as follows:

1. A child offender: aged below 18.
2. A youthful offender: aged between 18 and 21.

### 1.2. Juvenile Delinquency and Young Prisoners in Malaysia

Sidhu [7] reported an increase in the index crime statistics in Malaysia in 2004. According to him, in the 1980s and 1990s, crimes in Malaysia only involved minor robbery cases with minimal physical injuries. Over time, criminal activities became more sophisticated and complex. Syndicated and white-collar crimes involving local and international transactions have become popular and are more complicated for the authorities to handle. “There is an increase in crime from 70,823 in 1980 to 156,455 in 2004. This amounts to an increase of 120%. Similarly, violent crime has grown by 192% and property crime by 112% during these two periods. Violent crime has increased by 57.4% and property crime by 15.4%. This shows that violent crime is growing at a faster rate than property crime” [7].

As of 21 October 2013, the number of young prisoners (under 21 years old) detained in ordinary prison institutions was a total of 2058 people, of which 1168 people had been convicted and 890 people had not been convicted (remand detention). The total of 2058 represented 5.5% of the total number of inmates detained in regular prison institutions at that time. As of 21 October 2013, a total of 11 teenagers (under 21 years old) were detained at the Moral Rehabilitation Center of the Prison Department. The number of teenagers (Children Act 2000) detained at the Henry Gurney School institution, Malaysian Prison Department, was 886 people.

If we added up all the young prisoners, young citizens, and juveniles who were in regular prison institutions (the Moral Rehabilitation center and the Henry Gurney School), the total number of young prisoners would have been 2955, which represents 7.3% of the total prisoners (40,089) in all prison institutions in Malaysia. To be more detailed, the number of convicted young prisoners and young citizens in remand was 4.5%, and the number of convicted young prisoners alone was 4.1% of the total number of citizen inmates [8].

### 1.3. Influences of Family Life and Peer Pressure on Delinquency

Previous researchers have identified several factors that contribute to the growing number of crimes among the youth in Malaysia. These include aggression [9,10], poverty [11], and family structure [12], among others. Juvenile delinquent behaviors are influenced not only by their environment, but also through their observations. Juveniles learn things based on what they hear and see from their parents, friends, and society at large. Chung and Steinberg [13] argued that “Juvenile delinquency is not an inherent human condition, but rather is learned through association, imitation, observation, pressure, needs, wants, influence and desires”.

In addition to this, Ezarina, Fauziah, and McWhirter suggested that family economic hardship and peer pressure were the main factors that contribute to youth being classified as at-risk [3,4,14]. They also found other related factors, which included single-parent families, as well as parents with low education backgrounds. With such underprivileged backgrounds, these youths are more likely to experience school failure and other negative outcomes. As a result, they might get involved in various unhealthy activities, such as loafing, illegal racing, and other social problems.

The loss of love and parental guidance at the basic levels of marriage and family also has broad social consequences for adolescents and society. Previous researchers [15,16,17,18] have indicated that too many young men and women from broken family institutions tended to have a lesser sense of connection with their neighborhood and are prone to making use of its members to satisfy their unnecessary needs or desires. The loss of a sense of belonging with the local community, as well as the isolation from the neighborhood, result in social chaos and various criminal behaviors. Apart from that, without proper supervision from parents, teenagers might get involved with the wrong people, who teach them to engage in unwanted behavior.

As with family, peer behaviors have a significant impact on teenagers’ personalities, attitudes, and ideologies. Peers basically have more influence during adolescence than at any other stage in life [19]. The socialization process shapes an individual’s perspective and character because we all learn, and usually adopt, the norms of our culture through socialization activities. Teenagers in particular like to share problems and seek help from friends when faced with various situations and obstacles in life. Indeed, Samsudin [20] found that, in general, youth spend approximately 16.3 h per week loafing [21]. The hours of loafing increased with age; those aged 22–25 years loafed around 18.8 h per week, as compared to those aged 12–15 years, who loafed around 14.4 h per week. The number of hours that youth spent loafing also increased according to how the youth perceived themselves. Those who saw themselves as weak loafed more often than those who perceive themselves as excellent, with 26.3 h and 17 h, respectively.

Most property crime offenders are unemployed or have low-income jobs. Previous researchers have found a significant relationship between unemployment and property crime [15,21,22,23,24,25,26]. On top of that, Shong [11] found that, among juvenile offenders, poverty was linked to a miserable family environment, academic failure, and involvement with deviant friends.

### 1.4. The Need for a Study about the Influence of Family Life and Peer Pressure on Delinquency in Malaysia

From the review of previous research in the literature, it was found that many studies conducted in Malaysia had identified problem-solving skills, low socioeconomic status, gender differences, and dropping out of school as factors in criminal behavior. Other studies investigated the type of crime. Few studies have been conducted combining the influences of both family role and peer pressure on delinquent and criminal behavior.

In addition, previous findings and arguments forwarded by international scholars might be insufficient for representing the local context due to differences in cultural values, lifestyle, religion, and geographical features [27,28,29]. Studies on factors that contribute to criminal behavior among young prisoners in Malaysia are deemed important for gaining more information on the issue. These factors may serve as guidance for future researchers and authorities in Malaysia to develop modules in schools, which can help prevent teenagers (or school students) from becoming involved in various types of crime and negative behaviors. An in-depth understanding of the delinquent behavior issues will serve as a baseline for the implementation of preventive approaches and measures to minimize social issues in Malaysia. Therefore, the present study aims to fill the research gap and implies the importance of understanding the behavior for parents.

## 2. Materials and Methods

### 2.1. Research Design and Participants

A qualitative research design is involved in establishing answers to the whys and hows of the phenomenon in question. The study, qualitative in nature, used a multiple case design with purposive sampling of typical cases, which were selected considering the following *inclusion criteria*: age (adolescents under 21 years old); being deprived of liberty for the commission of a crime; not being aggressive; and voluntarily agreeing to participate in the study. Because the present study explored the influence of family life and peer pressure on delinquency and criminal behavior among young male prisoners, who are the hard-to-reach population in Malaysia, a qualitative data collection method was applied. Specifically, in-depth interviews were conducted using an interview protocol, which, to obtain information from the informants, comprised open-ended questions. Questions were validated by two independent subject-matter experts who had backgrounds in criminology and psychology. Each interview session averaged between 100 min and 120 min.

### 2.2. Ethical Consideration

Permission from the Malaysian Prison Department was obtained with written approval. All participants signed an informed consent form to protect their identity and to ensure the transparency of the sample selection process. All young male prisoners who participated in this study did so on a voluntary basis, under no pressure from prison officers or researchers.

### 2.3. Samples

To explore the role of family and the influence of peer pressure, a qualitative research design using an in-depth interview approach was applied. In total, 12 young male prisoners were selected for this study using a purposive sampling technique. Because this qualitative study was largely investigatory and relied on in-depth data, the present research utilized the concept of saturation as a basis for the sample size. The sample was selected with the following criteria: (1) male Malaysian young people (aged 9–21), who had committed crimes and were serving a sentence for one or more offenses; (2) Malaysian young people (aged 14–21), who were detained in correction institutions under the Malaysian Prison Department; (3) young male prisoners, who were under court order; (4) non-aggressive prisoners, who were not detained under certain preventive laws such as the Prevention of Terrorism Act (POTA) and the Prevention of Crime Act (POCA).

Table 1 summarizes the demographics of the sample. The age of the sample ranged from 16 years to 21 years old. The types of crime they committed included armed robbery (n = 2), murder (n = 2), drug-related crime (n = 2), hiding birth (n = 1), burglary (n = 1), robbery (n = 1), and rape (n = 3). The term of punishment varied from remand on custody to a maximum of 12 years. Six out of the twelve participants had been staying with only one parent prior to incarceration.

### 2.4. Data Collection

Unlike a normal conversation, the purpose of the interview was to gain further information pertaining to the study. Four types of questions were included. They were: (1) introductory questions; (2) questions that indicated transitions from one section to another; (3) questions that focused on the key aspects of the study; and (4) closing questions [30,31]. The number of meetings with each participant varied from one to two, depending on the responses. Some participants were contacted twice to clarify certain information and details. Two interviewers were present during each interview and important observations and notes were taken. We used focus groups combined with interviews to research the participants’ experiences and how family and peers had influenced their behaviors. A combination of both methods enhances data richness and provides more in-depth insights into the phenomenon [32].

In-depth interviews were conducted to explore the role of family members and the influence of peer pressure. As such, interview questions were designed based on the literature reviews and research questions related to these issues. An interview guide was prepared, but additional questions were also asked depending on the responses of the participants.

The participants were asked the following questions during the in-depth interview: “is any of your family members committed to the crime?”, “how is your relationship with your family members?”, “can you describe about your family?”, “how is your relationship with your peers?”, *and* “in what ways, you are influenced by your friends?” As mentioned earlier, for the principle of preserving their reliability and validity, the interview questions were reviewed and validated by the two subject-matter experts before the actual data collection process.

To ensure data saturation, the interviewer investigated the topic of interest with the participants until there was nothing left to add. For example, this was done by using questions at the end of the interview such as “Anything else?” or “Do I need to know anything other than what I have asked you?” This was done to ensure that saturation had been achieved—that there was nothing else to add to the topic of interest. Whereas for credibility, the researchers used the triangulation of sources. The participants were interviewed at different points in time and participants with different perspectives were compared. The data were saturated at the 10th informant, as there were no new themes emerging after 10 informants. Additionally, two more informants were included to ensure that the data were well-saturated. Because the nature of the qualitative study focuses more on obtaining an in-depth understanding on the topic, this study was therefore concluded with 12 informants after considering the saturation/richness of the data we had obtained.

### 2.5. Analytic Strategy

A thematic analysis was employed to examine the influences of family life and peer pressure on delinquency and criminal behavior among young male prisoners in Malaysia. The thematic analysis method was chosen as it is widely acknowledged as the most common form of analysis in qualitative research. The thematic analysis includes identifying themes, concepts, and meanings [33]. The analytic process involves thorough transcription work. After a clear reading of the interview transcripts, categorizations of themes and keywords were developed, revolving around how the effects of family role and peer pressure on delinquency among young male adolescents in Malaysia were framed.

## 3. Results

The findings of the present research revealed several themes, under the aspect of family life, which related to the youngsters’ involvement in crime. The identified themes were as follows: (1) lack of affection from the family; (2) problematic family; (3) revenge on family members; (4) family members’ involvement in crime; (5) peer pressure; and (6) being involved with a criminal gang.

### 3.1. Lack of Affection from Family Members

The lack of love and affection was one of the factors that contributed to the criminal involvement among the young men in the sample. The findings indicated that eight participants (66.7%) who were involved in crimes did not receive enough affection from family members and parents. Teenagers who feel more affection-deprived tend to feel lonely, unhappy, and are more likely to experience depression and stress, which might lead to serious problems such as emotional imbalance and the development of criminal behavior.

The lack of affection and attention from family members had made most of these young prisoners feel unwanted and unloved by their family members. Participant 6, for example, stated that he was marginalized by the family members because he was the only adopted child. Participant 3 addressed that he did not receive any attention from his family members.

*My relationship with family members is not special. My mother loves my older brother more than she loves me*

(Participant 6)

*My family does not have time for me and listen to my problems. They don’t bother about my presence*

(Participant 3)

Based on the above excerpts, it can be observed that skills and lack of affection from family members have been identified as factors contributing to the young people’s involvement in crime. Teenagers who do not communicate with parents normally spend more time with friends and, sometimes, with bad people who influence them to take part in dangerous and negative activities.

### 3.2. Problematic Family

The second factor that contributed to the youngsters’ involvement in crime was having a problematic family. A significant number of interviewees described problematic relationships with their parents. A total of eight out of twelve participants (66.7%) claimed to have various family conflicts. Divorced and/or irresponsible parents had become factors that led them to delinquency, resulting in these young men being sent to prison. Participant 2, for example, stated that there was a constant argument and fight between his parents, because his father was suffering from mental illness. He claimed that:

*When I was little, baba (father) and mum (mother) like to quarrel. Baba is having a mental illness. Baba always grabs a knife and sits near to mum and baba always was admitted to a mental hospital. Baba will only become normal if he takes medicine*

(Participant 2)

Many parents were busy working to earn more money and support their families. Due to the increasing cost of living, parents must work hard to make ends meet. As a result, parents tended to fail to fulfil another crucial role that they have to play—educating their children. Busy parents and distant relationships among siblings were also factors that contributed to crime among youngsters. Participant 7, for example, claimed that his parents were too busy with their own problems.

*The family does not care, both father and mother are busy, and all family members do not like me because I’m a bad person. Dad always hit me, and my parents separated when I was thirteen*

(Participant 7)

Participant 11 also claimed that he was living a hard life with his mother, who was a single parent:

*I was born in a very poor family. I live with my mother after she divorced my father. I did not see my dad since I was three*

(Participant 11)

Participant 12 also gave the same response when he was asked about his family life. The young prisoner shared his experience living with abusive siblings and untruthful parents. According to him, his elder brother once stabbed his father in front of his eyes.

*My family is troubled. My dad has another woman and my brother stabbed my dad*

(Participant 12)

### 3.3. Revenge on Family Members

The urge to take revenge on family members was the third factor that contributed to the chances that young people would be involved in crimes. The findings indicated that 10 out of 12 participants (83.3%) claimed that they committed crimes due to the urge to seek revenge on their own family members. Participant 1, for instance, had a deep grudge against his father for being abusive toward his mother. He said,

*I live with a vengeance for my own father. He once hit me with a baseball bat. I feel like killing him all the time, I even kept a knife under my pillow. I have tried to kill him once, but my mother found out about it when she saw the knife*

(Participant 1)

Both Participants 3 and 4 hated their brothers due to some trust issues. Participant 4 shared the following story:

*I hate my elder brother as my parents paid special attention to him. He was always treated differently than me. I felt neglected. I thought if I kill him, I might get attention. I tried to kill him once but failed. My elder brother told other family members about some bad activities that I committed, when I was 15*

(Participant 4)

Participant 10, on the other hand, had no solid reason to hate his family members, but he still did. He said,

*I hate my brother and all my family members. There is no specific reason why I hate my family members as I have never felt happy with my family members. I don’t know why, but I just feel not satisfied with them*

(Participant 10)

Participant 11 hated his father for neglecting the family when they were going through a hard time. His family was abandoned by the father after the divorce.

*Even when my father was with us, he never used to take care of us. He is so concerned with his own entertainment and happiness. I was angry with him. But my anger grew with my dad when he never came to see us and let us live a hard life after separation. Our life was very hard, and it is all due to him*

(Participant 11)

### 3.4. The Involvement of Family Members in Crime

The involvement of family members in crime was also seen as one of the factors that contributed to the development of criminal behaviors among the young male prisoners involved in this study. Six out of twelve participants (50%) claimed to have at least one family member who was involved in crime. Some of the family members of these participants were among the individuals who had a criminal histories in Malaysia. Participant 1, for instance, claimed that his father had been jailed for running a drug-dealing operation. Participants 3, 4, and 8 also had family members with various criminal records. Participant 3 shared the following story:

*My brother was a drug dealer, while my other brother was an alcoholic involved with hookers and prostitution activity*

(Participant 3)

Participant 9, who was charged with murder, claimed that one of his family members was also serving his punishment in jail due to the same crime.

*One of my family members was arrested and charged with murder, so did I*

(Participant 9)

### 3.5. Peer Pressure

Peer pressure is exercised when a teenager decides to attempt something in order to be accepted and valued by his friends. This pressure involves acting on deviant actions against his will or beliefs. In this study, eight of twelve participants (66.7%) claimed that friends played a very important role in their life. Participant 1 described his friends as saviors who had never failed to help him every time he got into trouble.

*My friends have a great impact in my life. I always trust them more than my family. They are the savior of my life because they have helped me when I was in trouble. My friends are part of my life*

(Participant 1)

Adolescents are always curious to know about the opposite sex. Neither parents nor teachers here were ready to clarify their doubts about sex. Usually, adolescents seek the help of their friends in this regard, which, most of the time, leads to unhealthy sexual acts. Participant 2 stated that his life changed when he met his friends; They also taught him many things, such as how to have sex.

*I went through many changes after I met my friends. They are the ones who taught me about sex and clarified my doubts about intercourse. They told me some dark secrets. We went through a lot of things together. I have been with them since I was 13*

(Participant 2)

Participant 4 claimed that his friends were a lot older than him. Mature friends had influenced his life and changed his perspective in so many different ways.

*I’m stuck with friends who are older than me because they are more mature than me. And they have a better idea about everything compared to my friends of the same age. At times I look at them with a feeling of awe. This made me learn many things from them. I have learned a lot from them including various techniques in crimes*

(Participant 4)

A total of 75% of the participants involved in the present study admitted that their criminal behavior was influenced by friends.

### 3.6. Peers from Criminal Gangs

Five out of twelve participants admitted that they had been involved in activities such as smoking, drinking alcohol, prostitution, and using illegal drugs when they were with a gang of friends. Participant 1 admitted that he took drugs and robbed every time he felt bored, and that he usually did that with his gang of friends.

*I usually take drugs such as cannabis. I take it from my friends who sell drugs. I go to bed whenever I feel bored. After we (friend’s gang) get the money, we’ll go to the club to enjoy it*

(Participant 1)

Participant 3 stated that he usually went to a nightclub with friends for a drink and to hire a prostitute. Participant 7 stated that smoking and nightclubbing were among the regular activities he did with his friends. Both Participants 8 and 11 shared the same experience. In addition to shopping and watching movies, prostitution and drug addictions were among their favorite activities when they got together with friends.

## 4. Discussion

### 4.1. Influence of Family Life on Delinquency

Children require affection in their psychological development process. In other words, all the love and affection that a child receives in his childhood will be reflected in his or her psychological and emotional development. In the present study, it was found that family conflict, divorce, family separation, mental illness in the family, domestic violence, imprisonment, alcohol addiction, and prostitution were the main factors for delinquency and criminal behavior. The findings of the present study are consistent with the previous studies. Sabramani [24] identified a number of factors contributing to a violent crime, such as the increase in the number of school dropouts among young teenagers, poverty, unemployment, disrespect issues, as well as poor parenting skills, and little adult supervision. Zainudin and Norazmah [34] found that parents who did not spend much time at home usually had communication problems with their children. In addition, studies have proven that communication between parents and children have a connection with children’s positive behaviors, such as academic excellence, and children’s negative behaviors, such as drug abuse, alcoholism, and other misconduct [35].

Similarly, the marital relationship between husband and wife plays an important role in child development. Divorce, separation, and uninvolved parenting styles lead to unhealthy behaviors in the child. Our study is in line with another study conducted by Mohd Taib Dora and Ezarina [3,18]. In his study, he identified family conflicts as being one of the key factors that are likely to increase the chances that young people would become involved in crimes. Divorce, poverty, busy lifestyle, and abusive family members were also among the risk factors that increase the chances of younger people committing crimes.

Previous researchers have indicated that family conflicts have become included among the factors that increase the chances of young people committing crimes. Arieff and Wardah [36] described the attitudes and personalities of adolescents as being a result of emotional behaviors. Azizi and Rosnah [17] claimed that low self-control predicted a range of criminal behaviors due to intense feelings of hatred, dissatisfaction, and disappointment. This was again proven in our studies.

Regardless of their size or structure, the families of the participants involved in the present study experienced various issues that preceded the young people’s crime involvement. Teenagers need a healthy living environment to learn and grow up. According to Tan [12], poor family relationships lead to juvenile delinquency in Malaysia. It is clear that family is the foundation that helps develop this healthy living environment. A dysfunctional family with conflicts and issues brings a negative impact on a teenager’s emotions and well-being. Adolescents living in conflict, and who have psychological problems, are prone to becoming antisocial [4] and involved in various crimes and social problems. Previous studies have suggested that the basis of many crimes, globally, lies in the parents’ loss of ability to be responsible in taking care of their children. The current study highlighted the roles of parents and friends in predicting the involvement of young offenders in crimes.

### 4.2. Influence of Peer Pressure on Delinquency

Teenagers’ attitudes and personalities are shaped by wanting to feel that they belong to a group of friends or peers. Teenagers tend to turn to their friends whenever they feel unsatisfied with their family [11]. At this stage, friends become the strongest support in a teenager’s life; thus, getting friends’ approval in many things remains an utmost priority. According to previous studies, the current study showed that friends have a very strong influence on an individual’s behavior [11,14,16].

Past research has explained that reinforcement plays an important function in the influence of peers. At the adolescent stage, the importance of peers increases and adolescents look to their peer group for direction on the appropriate ways of thinking and behaving. Although reinforcement is dependent on the individual’s ability to encode and interpret social cues from their peer group, if an adolescent perceives a favorable response from peers for deviant behavior, then they are more likely to engage in that particular behavior. However, they need not even observe delinquency directly, they only need to believe that the behavior will elicit this approval [37].

Gottfredson and Hirschi [38] suggested that the time spent socializing with the wrong influencers, with very minimal parental guardian involvement, contributes to criminal behavior and law-breaking activities. The development of strong and positive social ties prevents individuals from participating in negative activities. The study by [39], which involved 210 university students, found a significant relationship between social skills and peers with delinquent behavior. This explains that a socially adept person is unsusceptible to delinquent behaviors [40].

## 5. Implications of the Study

The implication of this study for parents is educating them on the fact that their presence is very important for guiding and teaching adolescents, and to prevent them from getting caught up in delinquent behaviors. Through this study, parents can find out how important it is to educate adolescents from an early age with the right type of education and can, in turn, reduce the problem of crime and delinquent behavior among adolescents. At the same time, the findings of this study will raise society’s awareness that parents play an important role in the formation of adolescents, the fact being that society itself also plays a serious role in addressing the problem of social symptoms that occur among adolescents. Society also plays a role in not cursing and looking askance at teenagers who commit these offenses, instead providing help and guidance to return them to the right path.

Additionally, this study is also suitable as a guide for developing modules in schools to prevent adolescents and school students from engaging in crime and delinquent behavior. It is also suitable as a guideline for teachers to guide these students and adolescents toward the formation of superior personalities and become individuals who can succeed brilliantly. The findings of this study also indicate that the role of family is a significant factor for young people who are involved with crimes. Family members interact with one another as a system. Thus, the problems of any one family member can significantly influence the other members. Families face multifaceted problems. Although the problems are usually classified, they exist as family problems and as one extensive system. Criminal-related issues are a common problem faced by young people. Among other factors that contribute to youngsters’ involvement in crime is a dysfunctional family. As a result of the family’s dysfunction, the children’s education is also neglected, causing the problem of dropouts from the formal education system. Families should maintain a state of equilibrium and any crisis regarding dysfunctional roles requires specific intervention, particularly from family social workers and community outreach workers.

In this regard, family social work is a helping profession that professionally intervenes in families with criminal-related issues. The family social worker applies a generalist approach in working with at-risk families. The concern encompasses many different types of programs, such as preservation services, in-home family support, and enhancing parenting skills. Regarding the family social worker involved with delinquent family members, family social work practices embrace the following objectives: (1) evaluating the psychosocial aspects of the family by performing the needs assessment; (2) preparing the family for long-term intervention phases by reinforcing strength; (3) creating substantial changes in family functioning to maintain independent routines; (4) helping families maintain effective social functioning by providing adequate professional supports; and (5) addressing family crises, so that they can be effectively managed and predicted. In some situations, social work with families may go beyond the family system and involve work with the immediate neighborhood or the community. Therefore, this study is also suitable as a guide to develop family social work and community empowerment modules to prevent adolescents and the at-risk community from engaging in crime and delinquent behavior.

## 6. Conclusions

The findings of the present study indicate that the role of family and peer pressure are significant factors that increased the probability of younger people committing crimes. Therefore, despite its limitations, the current study implies the importance for parents to get to know their children’s friends and the people around them. Parents are advised to spend some time with their children every day to understand them more. This is because parents are the ones who are closer to them. This study also implies the importance of guidance and counseling for parents, teens, and teachers. Furthermore, this paper also serves as a suggestion for policymakers to evaluate how well current policies or modules work to prevent more criminal activities from being committed by young prisoners in the future. In addition to that, this study offers an in-depth understanding of the delinquent behavior that underpins the baseline of the implementation of preventive approaches and measures to mitigate social issues in Malaysia.

From the public health point of view, it is essential for a single agency in Malaysia to play the proactive role, to take charge in uniting all other relevant agencies, including public, private, and non-governmental organizations, and to push for the implementation of policies that target family reform in the country. Due to the diverse administrative ecosystem in Malaysia, organizations are working in isolation towards addressing juvenile delinquency. Due to this, we are lacking much needed support in terms of expert opinions, financial allocations, and innovative approaches for addressing this issue. Thus, the impact for change is not strong due to lack of strong leadership. This is especially clear in the formation of Malaysia Council of Tobacco Control (MCTC). It is an organization consisting of more than 40 organizations, united in tobacco control efforts. The recent breakthrough announcements by the Malaysian Government related to smoking were contributed by the council’s tireless effort, which created the much-needed impact and pressure for government to announce the change.

A united organizational effort benefits society. A solitary financial budget can be obtained to further strengthen and improve the current infrastructure. It includes training more personnel to ensure availability of experts, producing updated materials, utilizing technology to address these issues, implementing impactful nationwide mental health programs and clear standard guidelines for post-intervention impact measurements, and improvising to ensure the sustainability of any strategies. Furthermore, it is easier to convince the current leaders to recognize the hidden threat of juvenile delinquency by giving it greater emphasis.

Moving forward, as we recognize the usage of surveillance data for communicable disease control, juvenile delinquency must be further controlled by implementing behavioral surveillance. With such an approach, data can be obtained to monitor the incidence much more closely. Timely and immediate interventions can be implemented from the data. This approach is based on our understanding that, in matters related to behaviors, it is an ongoing process and it is impossible for a single point of intervention to prevent further occurrence. Innovative ways of using computer applications are much needed. Going forward, an algorithm for the application of artificial intelligence in predicting different aspects of juvenile delinquency could generate new breakthroughs for us to curb this highly complex illness.

More studies pertaining to the same issues and concerns but using different groups of participants, are recommended, as are studies using participants involved in different types of crimes and social issues. This study also helps future researchers to understand the effects of family relationships and peer pressure among teenagers in general, specifically among young offenders. By understanding the factors that emerged in the present study, they, along with other related factors (such as the absence of a strong father figure, lack of maternal love, parenting style, family size, house or neighborhood condition, peer group age, and more) can be explored to see how far they can influence teenagers to break the law.

## 7. Limitations and Future Research

Such as in other research, this current study is not without limitations. The first limitation is that this research only represents a specific group of young male prisoners in Malaysia (mostly Malays). Therefore, the findings may not be applied to other ethnic groups and cultural contexts. Although they are consistent with existing research, due to this small sample size, our findings may not be generalized. Last, but not least, we focused only on male participants. Hence, our findings may not apply to young female prisoners in Malaysia.

## Figures and Tables

**Table 1:** Participant Background Demographics.

P	Age	Ethnic Group	Father’s Occupation	Mother’s Occupation	Education Level of Informants
1	16	Malay	Passed away	Baker	Upper secondary
2	20	Chinese	Business	Not working	Lower secondary
3	19	Malay	Pensioner	Cleaner	Primary
4	19	Malay	Securities	Not working	Upper secondary
5	19	Malay	Driver	Not working	Lower secondary
6	19	Malay	Department of Veterinary Services	Not working	Vocational college
7	19	Malay	School assistant	Clerk	Upper secondary
8	20	Malay	Army veteran	Clerk	Upper secondary
9	20	Indian	Passed away	Work in Singapore	Upper secondary
10	20	Malay	Passed away	Taxi drivers	Primary
11	21	Bajau	Narcotic Dept. in Sabah	Not working	Lower secondary
12	20	Malay	Factory supervisor	Cleaner	Primary

## Data Availability

The data presented in this study are available on request from the corresponding author. The data are not publicly available due to data confidentiality imposed by the Malaysian Prison Department.

## References

[1] Chaffin M., Silovsky J.F., Funderburk B., Valle L.A., Brestan E.V., Balachova T., Jackson S., Lensgraf J., Bonner B.L. (2004). Parent-child interaction therapy with physically abusive parents: Efficacy for reducing future abuse reports. J. Consult. Clin. Psychol..

[2] Kruttschnitt C., Ward D., Sheble M.A. (1987). Abuse-Resistant Youth: Some Factors That May Inhibit Violent Criminal Behavior. Soc. Forces.

[3] Ezarina Z., Norulhuda S. (2019). Family Dysfunction of Out-of-Control Teenage Girls: A Preliminary Study. Glob. J. Al-Thaqafah GJAT.

[4] McWhirter J., McWhirter B., McWhirter E., McWhirter R. (2013). At-Risk Youth: A Comprehensive Response for Counselors, Teachers, Psychologists, and Human Professional.

[5] Kupersmidt J., DeRosier M., Dodge K., Kupersmidt J. (2004). How Peer Problems Lead to Negative Outcomes: An Integrative Mediational Model. Children’s Peer Relations: From Development to Intervention.

[6] Children Statistics, Malaysia, 2021, Department of Statistics Malaysia, Official Report. https://www.dosm.gov.my/v1/index.php?r=column/cthemeByCat&cat=333&bul_id=d1YxK0tsUWp4RGNHQXZTZTIzNUxWdz09&menu_id=U3VPMldoYUxzVzFaYmNkWXZteGduZz09.

[7] Sidhu A.S. (2005). The rise of crime in Malaysia: An academic and statistical analysis. J. Kuala Lumpur R. Malays. Police Coll..

[8] Malaysian Prison Department (2014). Program Pembangunan Insan.

[9] Mohammad Rahim K., Azizah O., Khaidzir I., Geshina A.M.S. (2016). Aggression Profiles of Incarcerated Malaysian Male Murderers. Akademika.

[10] Abdullah H.A., Adriana O., Norbaya A., Syamsyihana G. (2015). Aggressive and Delinquen2t Behaviour among High Risk Youth in Malaysia. Asian Soc. Sci..

[11] Shong T.S., Abu Bakar S.H., Islam M.R. (2018). Poverty and delinquency: Aqualitative study on selected juvenile offenders in Malaysia. Int. Soc. Work.

[12] Tan B.P., Zuriani J.O., Noor Banu M. (2017). Structure or Relationship? Rethinking Family Influences on Juvenile Delinquency in Malaysia. Asia-Pac. Soc. Sci. Rev..

[13] Chung H.L., Steinberg L. (2006). Relations between neighborhood factors, parenting behaviors, peer deviance, and delinquency among serious juvenile offenders. Dev. Psychol..

[14] Fauziah I., Ezarina Z., Salina N., Norulhuda S., Nazirah H., Siti Mariam M. (2020). Social pressure among former drug addicts after discharged from drug rehabilitation centre. Int. J. Psychosoc. Rehabil..

[15] Ha O.K., Andresen M.A. (2017). Unemployment and the specialization of criminal activity: A neighborhood analysis. J. Crim. Justice.

[16] Esiri M.O. (2016). The influence of peer pressure on criminal behaviour. J. Humanit. Soc. Sci..

[17] Azizi Y., Rosnah B. (2007). Punca Berlakunya Masalah Gejala Gengsterisme di Kalangan Remaja di Beberapa buah Sekolah Menengah di Empat Buah Negeri.

[18] Mohd Taib D. (2004). Isu dan Faktor Penderaan Kanak-Kanak dan Juvana di Negeri Johor Darul Takzim.

[19] Steinberg L., Monahan K.C. (2007). Age differences in resistance to peers. Dev. Psychol..

[20] Samsudin A.R. (1994). Tingkah Laku Lepak di Kalangan Remaja: Kajian di Semenanjung Malaysia.

[21] Abdul Aziz M.H. (1987). Pengangguran Merupakan Faktor Utama Menyebabkan Peningkatan Jenayah Pecah Rumah di Daerah Temerloh. Unpublished Diploma in Police. Sciences Dissertation.

[22] Ahmad Ragib M.S. (1987). Pengangguran Merupakan Faktor utama Menyebabkan Peningkatan Jenayah harta Benda di Negeri Pahang. Unpublished Diploma in Police. Sciences Dissertation.

[23] Mohd Sabri M. (2002). Pengangguran dan Jenayah: Satu Kajian Keatas Banduan Dipenjara Sg. Buloh. Unpublished Diploma in Police. Sciences Dissertation.

[24] Sabramani V., Idris I.B., Ismail H., Nadarajaw T., Zakaria E., Kamaluddin M.R. (2021). Bullying and its associated individual, peer, family and school factors: Evidence from Malaysian National Secondary School Students. Int. J. Environ. Res. Public Health.

[25] Andresen M.A., Linning S.J. (2016). Unemployment, business cycles, and crime specialization: Canadian provinces, 1981–2009. Aust. New Zeal. J. Criminol..

[26] Nordin M., Almén D. (2017). Long-term unemployment and violent crime. Empir. Econ..

[27] Mohammad Rahim K., Rohany N., Wan Shahrazad W.S., Rozainee K., Zainah A.Z. (2017). Validity and Psychometric Properties of Malay Translated Religious Orientation Scale-Revised among Malaysian Adult Samples. Akademika.

[28] Mohammad Rahim K., Rohany N., Wan Shahrazad W.S., Sarah Waheeda M.H., Janetter M., Nur Atiqah A., Rozainee K., Zainah A.Z. (2018). Validity and Reliability of Malay Version Financial Well-Being Scale among Malaysian Employees. Akademika.

[29] Lee E.H., Mohammad Rahim K., Norruzeyati C.M.N., Hilwa A.M.N., Noremy M.A. (2019). Exploring the psychometric properties of Mandarin-translated Zuckerman Kuhlman personality questionnaire among Chinese high school students in Malaysia. Int. J. Recent Technol. Eng..

[30] Creswell J.W. (2007). Qualitative Inquiry and Research Design: Choosing among Five Approaches.

[31] Castillo-Montoya M. (2016). Preparing for interview research: The interview protocol refinement framework. Qual. Rep..

[32] Lambert S.D., Loiselle C.G. (2008). Combining individual interviews and focus groups to enhance data richness. J. Adv. Nurs..

[33] Braun V., Clarke V. (2006). Using thematic analysis in Psychology. Qual. Res. Psychol..

[34] Zainudin S., Norazmah M.R. (2011). Faktor-faktor yang mempengaruhi remaja terlibat dalam masalah sosial di Sekolah Tunas Bakti, Sungai Lereh, Melaka. J. Educ. Psychol. Couns..

[35] Hartos J.L., Power T.G. (2000). Association between mother and adolescent reports for assessing relations between parent-adolescent communication and adolescent adjustment. J. Youth Adolesc..

[36] Arieff S.R., Wardah M. (2006). Membentuk Jati Diri Remaja.

[37] Miller H.V., Jennings W.G., Alvarez-Rivera L.L., Lanza-Kaduce L. (2009). Self control, Attachment, and Deviance among Hispanic Adolescents. J. Crim. Justice.

[38] Gottfredson M.R., Hirschi T. (1990). A General Theory of Crime.

[39] Wan Su H. (2008). Hubungan Personaliti, Kemahiran Sosial, Kebimbangan dan Rakan Sebaya Dengan Tingkah Laku Devian di Kalangan Pelajar Universiti.

[40] Rathakrishnan B., George S. (2021). Gambling in Malaysia: An overview. BJPsych Int..

